# Dr. D. M. Murray

**Published:** 1912-08

**Authors:** 


Dr. D. M. Murray, 32 years old, of Brooklyn, N. Y., a prominent physician of that city, was drowned at Reeb's Bay, just west of Port Colborne, on Sunday, July 21.

